# Study of Two Constraints Impacting Measurements of Human Glycemia Using a Microwave Sensor

**DOI:** 10.3390/bios11030083

**Published:** 2021-03-15

**Authors:** Mohamed Amine Zidane, Hichem Amar, Amar Rouane

**Affiliations:** 1 Institut Jean Lamour, Lorraine University (CNRS-UMR 7198), F54000 Nancy, France; amar.rouane@univ-lorraine.fr; 2 Research Center in Industrial Technologies, RCIT, DZ19000 Setif, Algeria; h.amar@crti.dz

**Keywords:** diabetics, microwave sensor, tissue properties, vein, SAR, glycemia

## Abstract

The measurement of glycemia is impacted by several constraints; those constraints have to be identified and quantified when designing an electromagnetic noninvasive sensor. The second phase concerns the level of the influence of these constraints. In this work, we investigated the impact of vein radius located in the forearm on a resonant microwave sensor to measure glycemia. We performed a numerical simulation using COMSOL Multiphysics of a proposed tissue model that was in contact with a microwave resonator. Some other factors affect the measurement, such as temperature, perfusion, sensor positioning and motion, tissue heterogeneity, and other biological activity. The sensor must be robust to the above-mentioned constraints. Because vein size changes from one person to another, the dielectric properties seen by the sensor will be different. This has been demonstrated by the change created in the resonance frequency of the simulated sensor for different vein sizes. The second constraint that was assessed is the dosimetry. The specific absorption rate (SAR) of any electromagnetic device should be evaluated and compared with SAR limits in the safety standards to ensure the safety of the user. Simulation results are in good agreement with SAR limits in the safety standards.

## 1. Introduction

Diabetes is a worldwide issue that affects millions of people; it is a chronic disease that appears when the pancreas ceases to produce enough insulin, which is a hormone used for regulating blood glucose in the body. The global diabetes prevalence has been estimated to rise from 171 million in 2000 to 366 million in 2030 [1]. The usual consequences of diabetes are blindness, kidney failure, heart attack, stroke, and lower limb amputation. Therefore, it is very important to take effective measures to monitor and control diabetes and its complications. For this purpose, several studies on the development of noninvasive blood glucose sensors have been conducted; there are several different methods that have been exploited, such as ultrasound method [2,3], optical techniques [4,5,6,7,8], thermal methods [9], and electromagnetic (EM) methods [10,11,12,13,14], which emerged after some experimental results that demonstrated the correlation between dielectric parameters and blood glucose concentration [15].

Clinical performance of a low-cost near-infrared sensor for continuous glucose monitoring applied with subcutaneous microdialysis is proposed in [16]. The near-infrared spectroscopy sensor for glucose monitoring in serum is also studied [17]; another sensor that uses Raman spectroscopy is developed in [18]; Freedom Meditech developed a sensor based on measurement of lens autofluorescence, which can distinguish subjects with diabetes from those without [19,20]; Biovotion AG uses impedance spectroscopy combined with an optical technique [21,22]. Despite all of these manufactured products, not one has been marketed and sold.

Other works related to an EM method have emerged after some experimental results demonstrated the correlation between dielectric parameters and blood glucose concentration [10]. Some primary research was carried out in 2005 using an EM sensor based on eddy currents; then, extensive studies on the EM method appeared [10,11,12,13,14,23].

The sensor sensitivity in the study [23] showed its efficiency towards small changes in glucose concentration in water, which is a homogenous medium. However, the size of its sensitive face exceeds 2 × 2 cm, which results in a high tissue contact surface. Therefore, the sensor is exposed to a highly inhomogeneous tissue, and any small change in the tissue dielectric caused by others factors apart from glucose can be detected, which leads to a lack of selectivity. The microwave sensor that is used in this study occupies a smaller surface contact with the tissue in order to improve selectivity.

The microwave resonator sensor has been significantly developed recently based on the resonance frequency shift due to the blood glucose impact on its dielectric parameters [24,25]. Hence, the results of the above-mentioned studies encourage using an EM method. Nevertheless, the use of a sensor for the noninvasive measurement of blood glucose is very complicated. Indeed, some of the previous studies examined a homogeneous medium such as water or blood, but, in fact, the human body is a heterogeneous medium (skin, fat, blood vessels, blood, muscles, bones, etc.), and each medium has its own dielectric properties, which are different [26]. In addition, any modification of the medium size will introduce a change in the effective dielectric parameters. This is a major constraint that we cannot control; a small slip of the sensor at the level of the skin introduces a modification in the level of the effective permittivity seen by this sensor. This small move or vibration affects measurement accuracy.

Second, the effective thickness of the soft tissue layers changes with the pressure applied, which impacts the electrical coupling between the sensor and the skin; also, the blood is pushed away from the skin proportional to the pressure applied [27]. A solution has been proposed in [27] using a pressure-sensing circuit to estimate the pressure effect, which can help to improve the measurement accuracy of blood glucose. In addition, almost all of the research was conducted at standard laboratory temperature; there is not much research available on the combined effect of temperature and glucose level changes on the dielectric properties. Furthermore, the effect of temperature on various tissues is known to be frequency-dependent, with complex behavior [28]. Other factors affect the dielectric properties apart from the glucose, for example, some constituents found inside the blood, such as vitamins (ascorbic acid) and metabolites (uric acid). It has been demonstrated experimentally that maltose, fructose, and galactose produce only small changes in the dielectric properties, at least three times less than the effects of glucose [29].

Lastly, based on a person’s posture, body shape, and tissue distribution, it will be very complicated to associate the same sensor for every diabetic individual without identifying their tissue distribution. In addition, the volume of blood in the measured region affects the data of the sensor. The EM sensor radiates the GHz EM field onto the human—is their use safe? As in mobile phones [30,31,32,33,34], a dosimetry study must be considered. The amount of radiation energy to which the human body is exposed is estimated using the specific absorption rate (SAR). The SAR is determined at the highest certified power level in laboratory conditions.

For a phantom that is exposed to microwave radiation, its thermal biological effects is increased; as consequence, the absorbed power is related to the temperature rise and maintains the dosimetric indexes currently applied in biophysical research [32]. The electric field created by the microwave sensor is absorbed by the tissue. Due to the tissue’s high permittivity, most of the electric field is concentrated at its bottom side. It is extremely important to enhance the power delivered to the sensor to increase the energy that interacts with the tissue, thus improving the sensor sensitivity; however, there is a limitation in the input power that must be carefully controlled within the international regulations, and this is represented by the SAR. This limitation represents another constraint that should be studied.

The aim of this paper is to present a study of the simulation of two constraints that have not been considered yet in the literature, the impact of vein size on the sensor that is used on the forearm, and its local SAR for two proposed models. Experimental study of the vein is difficult to conduct on a human body (in vivo measurement) when the other constraints exist and can impact the sensor’s response. Moreover, measuring only the size of a living person’s vein experimentally and estimating its impact in real time on the sensor are not feasible unless the effect of vein size on the sensor response is known in advance.

## 2. Theoretical Study

The used microwave sensor in the following study is based on a circular split-ring resonator [35]. If we consider one SRR with a substrate of a known dielectric constant on one side and air on the other side, the resonant frequency f_1_ can be represented using Equation (1)

(1) $$f_{1}\propto \frac{1}{\sqrt{\frac{\varepsilon_{r1}+1}{2}}}$$

When a dielectric sample of the unknown dielectric constant is placed on the surface of the resonator, the resonant frequency changes to

(2) $$f_{2}\propto \frac{1}{\sqrt{\frac{\varepsilon_{r1}+\varepsilon_{r2}}{2}}}$$

By taking the ratio of Equations (2) and (1), we obtain the expression for the unknown dielectric constant of the sample as

(3) $$f_{2}=\frac{\sqrt{\varepsilon_{r1}+1}}{\sqrt{\varepsilon_{r1}+\varepsilon_{r2}}}f_{1}$$

In the case of this study, $\varepsilon_{r2}=\varepsilon_{effi}\left(\varepsilon_{muscle},\varepsilon_{blooed,}\varepsilon_{skin},\varepsilon_{vessel}\right)$, where $\varepsilon_{effi}(\varepsilon_{muscle},\varepsilon_{blooed},\varepsilon_{skin},\varepsilon_{vessel})$ is the effective initial dielectric parameter of the heterogeneous tissue seen by the sensor before introducing any change in the blood permittivity and vein dimension.

$\varepsilon_{effi}\left(\varepsilon_{muscle},\varepsilon_{blooed,}\varepsilon_{skin},\varepsilon_{vessel}\right)$ can be estimated using Equation (4).

(4) $$f_{2}\left(\varepsilon_{effi}\right)=\frac{\sqrt{\varepsilon_{r1}+1}}{\sqrt{\varepsilon_{r1}+\varepsilon_{effi}}}f_{1}$$

$f_{2}\left(\varepsilon_{effi}\right)$ is the resonance frequency when placing a model of forearm tissue on the sensor.

(5) $$f_{2}\left(\varepsilon_{effi},△\varepsilon_{g},△\varepsilon_{v}\right)=\frac{\sqrt{\varepsilon_{r1}+1}}{\sqrt{\varepsilon_{r1}+\varepsilon_{effi}+△\varepsilon_{g}+△\varepsilon_{v}}}f_{1}$$

where ${△\varepsilon }_{g}$ is caused by the blood glucose change and ${△\varepsilon }_{v}$ is the change caused by the different vein dimension. $f_{2}\left(\varepsilon_{eqi},{△\varepsilon }_{g},{△\varepsilon }_{v}\right)$ is the resonant frequency of the sensor after a change in the blood glucose and a change in the vein dimension occurred.

By taking the ratio of Equations (4) and (5),

(6) $$f_{2}\left(\varepsilon_{effi},△\varepsilon_{g},△\varepsilon_{v}\right)=\frac{\sqrt{\varepsilon_{r1}+\varepsilon_{effi}}}{\sqrt{\varepsilon_{r1}+\varepsilon_{effi}+△\varepsilon_{g}+△\varepsilon_{v}}}f_{2}\left(\varepsilon_{effi}\right)$$

Equation (6) describes how the vein in the forearm can affect the EM sensor response when measuring blood glucose.

## 3. The Dielectric Properties of a Simulated Tissue Model in the Forearm

In several studies, it is important to determine the tissue’s dielectric characteristic accurately for EM safety evaluations and to analyze, design, and develop a biosensor that interacts with tissue using an EM near field. Indeed, EM sensor technologies are constantly subject to new constraints and requirements, particularly with respect to their size, frequency, and sensitivity. The tissue’s dielectric characteristic was previously studied by the IT’IS foundation [26].

In this simulation study, we exploited their experimental dielectric results to simulate a model of tissue using COMSOL Multiphysics software. The sensor was placed on the forearm area, and it is composed of several modified split-ring resonators (SRR). A magnetic field was used to feed the resonators. It was generated by a coplanar waveguide. The power magnitude was chosen because the tissue constituents have a constant-relative permeability $\mu_{relative}$ of 1. A change in vein location and its size, as well as glucose change, have no effect on permeability.

**Figure 1:**
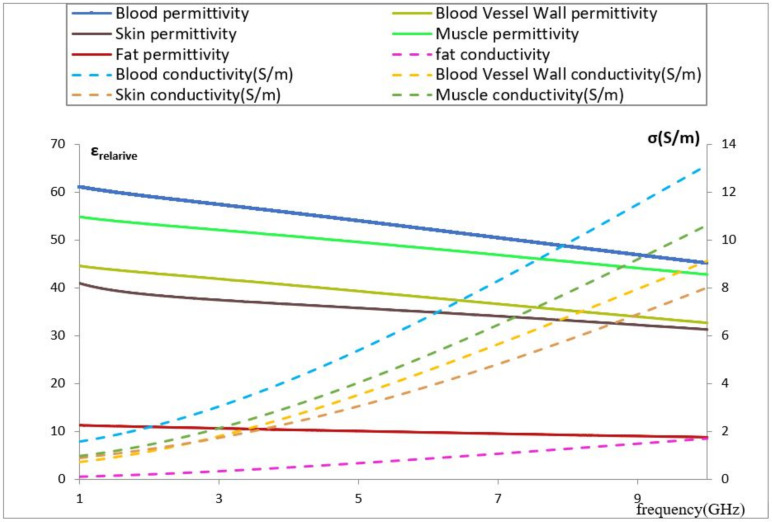
Permittivity and conductivity vs. frequency for different biological tissues [26].

Among the studies that are related to the development of a noninvasive blood glucose sensor, we identified some works related to the influence of volume, pressure on the EM sensor, and the impact of glucose on dielectric parameters in the study medium [28]. Nevertheless, we did not find studies related to the influence of vein dimensions and its location affecting the resonance. This occurs frequently with the human body due to its shape and its tissue distribution. Furthermore, it also depends on the body activity, which increases or decreases the blood flow and, subsequently, the vein radius can expand or shrink. This behavior, which will be studied, has an impact on the sensor and its SAR. Despite the current development of detailed computational models of specific organs, which are a prerequisite for specific applications, it is considerably difficult to simulate the anatomy of a real forearm considering its accurate dielectric parameters’ distribution because of its heterogeneous behavior. Furthermore, those dielectric parameters depend on the frequency for each tissue constituent (see Figure 1), which means to study any EM sensor based on the resonance frequency shift, it is very important to simulate the tissue model considering this dependency. All dielectric property data were imported from the IT’IS foundation database to our model tissue on COMSOL software.

Blood has a very high value of relative permittivity compared with other media due to its high water content, which means it contains higher energy storage in polarization and magnetization, and its value decreases as the frequency increases. Observing the above figure, we notice that blood introduces a conductivity higher than that of the other constituents at the same frequency. In relation to the impact of vein location and its dimension on our EM sensor, the high blood content of the vein will increase the specified absorption rate of the EM sensor, meaning that any small change in the vein dimension or location will impact the effective permittivity seen by the resonators.

## 4. Comsol Multiphysique Modeling

The numerical method used in the EM simulation is the finite element algorithm. The basic parameters used for the simulation are as described below.

The scattering boundary condition is presented by a cube (air block) that surrounds the antenna and the tissue model. The chosen option is no incident field, and the scattered wave type is a plane wave. This represents the EM wave propagating toward the outer space. The mesh size is normal. The reference impedance is 50 Ω. The type of the lumped port is multi-element uniform.

### 4.1. Model Description of an Area from a Thin Person’s Forearm

**Figure 2:**
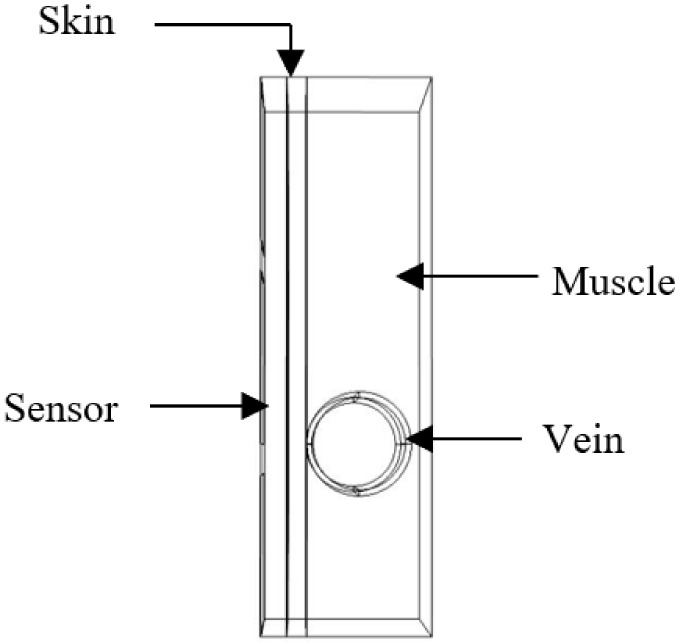
Part of the biological phantom on COMSOL.

The first proposed tissue model was obtained from a thin person’s forearm and simulated on COMSOL (Figure 2). The model consists of the following:

- Skin layer with a thickness of 1 mm;
- Blood vessel wall;
- Blood;
- Muscle.

The EM field produced by the microwave sensor penetrates into the blood through several layers with different dielectric parameters. In fact, any change in blood glucose impacts its relative permittivity and leads to a shift in the sensor resonance frequency, which is simulated by introducing a change in the relative permittivity of the blood.

During our simulation, a parametric sweep was carried out by introducing two variables. Δε, equal to 0.3, is a variation added to the initial relative permittivity of the blood to simulate a change due to glucose. The radius is the variable used to simulate the vein radius. The magnitude of the excitation voltage is 0.2 V.

### 4.2. Results and Discussion

**Figure 3:**
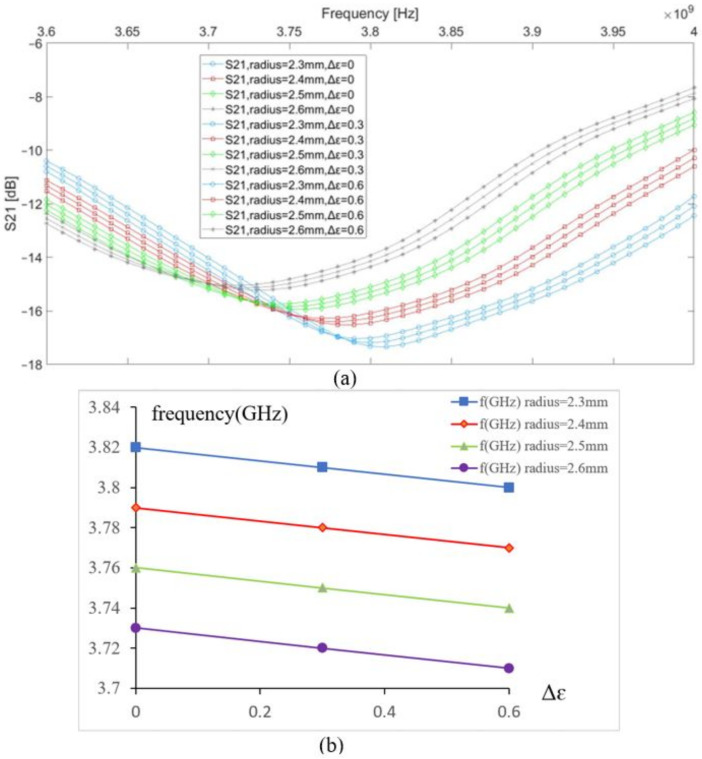
(**a**) S21 vs. frequency, (**b**) resonance frequency shift vs. permittivity change in the blood.

The vein radius was changed from 2.3 to 2.6 mm to study its impact on the resonance frequency based on the transmission parameter S21; meanwhile, we introduced a small variation into the blood permittivity by a step of 0.3 to simulate three different concentrations of blood glucose. Before introducing any change, the initial resonance frequency of the sensor in contact with the modeled tissue was 3.82 GHz. In Figure 3, the graphs in the same color have the same vein radius and different values of the blood permittivity; a shift in resonance frequency observed due to a change of 0.3 in the blood permittivity is equal to 0.01 GHz, and a shift in resonance frequency due to a change of the vein’s radius by 0.1 mm is equal to 0.03 GHz. This case occurs for any microwave sensor using a frequency shift technique. Any modification in the biological tissue’s composition details impacts its dielectric parameters, which affects the near-electric field coupling between the sensor and the body. The human body is a heterogeneous medium that dynamically evolves according to different factors; thus, it is essential to determine the influence factors on the tissue composition to optimize the coupling between the body and the sensor.

### 4.3. Model Description of an Area from an Overweight Person’s Forearm

**Figure 4:**
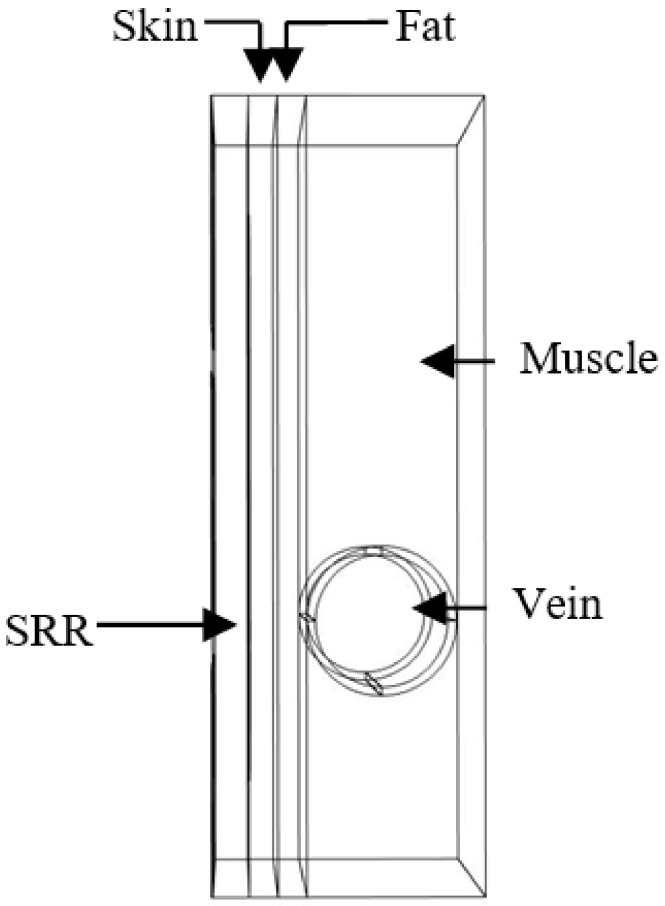
Simulated model of the forearm of an overweight person in contact with the sensor.

The second model was simulated by adding a fat layer with a thickness of 1 mm between the vein and the skin, which corresponds to an overweight person (see Figure 4).

**Figure 5:**
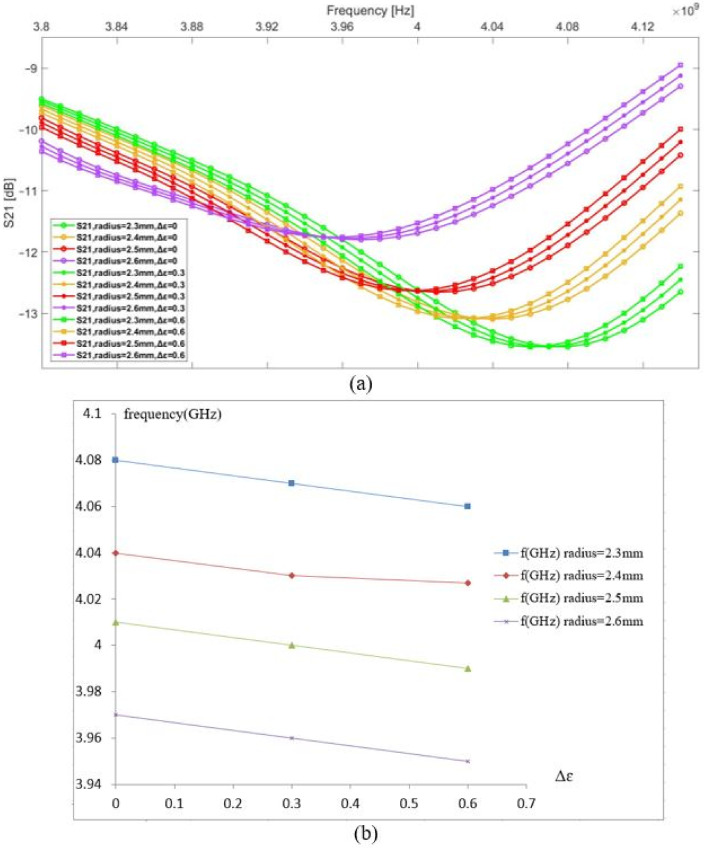
(**a**) S21 vs. frequency, (**b**) resonance frequency shift vs. permittivity change in the blood.

The dielectric parameters of the fat are presented in Figure 1. This tissue has a lower value than other biological tissue compositions (low water content), which favors the electric field infiltrating the fat layer toward the vein and muscles. In this simulation, the same study was performed. The initial resonance frequency is 4.07 GHz, which is higher than the previous model. Therefore, the penetration of the electric field is lower in this model. The graphs in the same color have the same vein dimension and different values of the blood permittivity. It was observed that a change in vein radius by steps of 0.1 mm introduces a 0.04 GHz shift in the resonance frequency for a change in the radius vein of from 2.3 to 2.4 mm and from 2.5 to 2.6 mm. However, a 0.03 GHz shift in the resonance frequency was noted for a change in the radius vein of from 2.4 to 2.5 mm (see Figure 5); this is due to sensor behavior. Indeed, the distribution of the electric field E inside the vein blood is not homogeneous; this distribution depends on the shape of the resonator.

By comparing both previously presented models, it is noted that any small change created in the vein dimension caused by pulse rate, temperature, activity level, and clothing, or even the different dimensions of the vein of two different people, affects the sensor measurements. These factors have an impact greater than glycaemia, and, thus, it is important to choose the sensor location where the volume of the blood remains almost constant. In addition, we must consider tissue distribution before making in vivo measurements.

## 5. Dosimetry

It is necessary to investigate the SAR of any radiating device and ensure compliance with the international commission on nonionizing radiation protection (ICNIRP) standard for health safety [34].

In the next section of this study, we evaluate the local SAR of our sensor using COMSOL software by considering the previous tissue models.

### 5.1. Methods and Model

#### Part of the Forearm Was Modeled Using COMSOL Multiphysics^®^ Software

To numerically evaluate the effects of exposure to radiation on the forearm, a previously used model that corresponds to overweight people was used. A fat layer of 1 mm was added between the skin layer and muscles with the vein. The model assumes that the dielectric properties of each tissue are spatially uniform and depend on the frequency.

The common method of calculating the estimated SAR value in COMSOL is by using the following formula:

(7) $$E_{SAR}=\sigma \frac{{|E|}^{2}}{\rho }$$

where σ is the conductivity of human tissue and ρ is the density $\left(E\right|$ is the norm of the electric field. The SAR unit is W/kg).

The boundary condition at the interface is given by

(8) n×E=0

### 5.2. Results and Discussion

COMSOL Multiphysics^®^ includes all necessary mathematical formulas to simulate EM wave propagation and combine it with a mathematical model of bioheat transfer. The numerical model presented here is based on the finite element method (FEM). In this simulation, the incident power used on port 1 of the sensor is equal to 1 mW, which is equivalent to 0 dBm. The resonant frequency of the proposed sensor in contact with the modeled medium is 4.06 GHz. The local SAR value was calculated using the total power dissipation density and the density of the different media.

**Figure 6:**
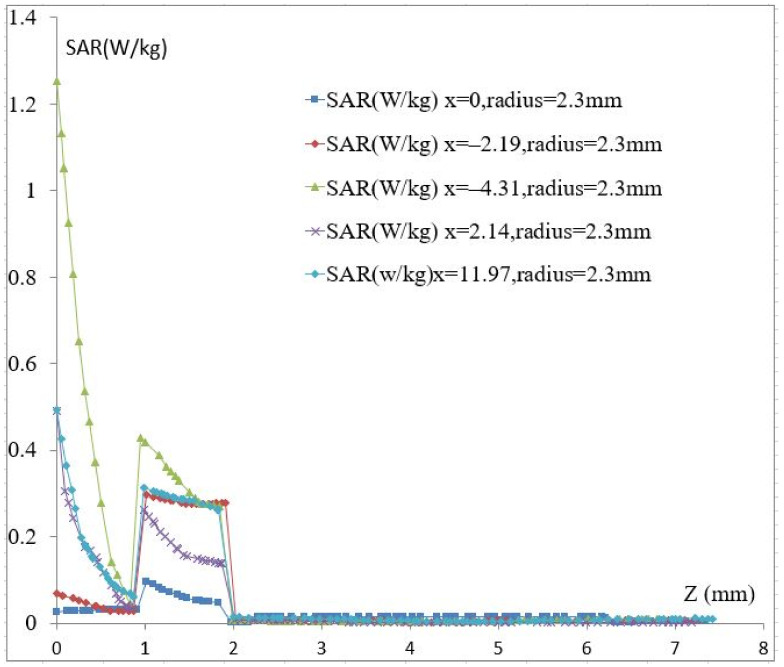
The calculated SAR. vs. Z.

The average SAR value must be less than 2 W/kg according to the ICNIRP standard for health safety in Europe [34] and less than 1.6 W/kg for uncontrolled environments as established by the US [35,36]. In this study, the vein radius is constant, and its value is 2.3 mm. Based on the SRR cell location, the obtained SAR maximum value is 1.23 W/kg. Figure 6 shows that skin and fat absorb almost all of the radiation. This is due to the different dielectric characteristics in the modeled part of the forearm; furthermore, the fat’s density is lower than that of the skin, which explains the higher SAR value at the 1 mm distance from the skin. We also observed a higher decrease in the SAR value in the skin layer compared with that in the fat layer. At x = −4.31 mm, the change in the SAR is equal to 1.17 W/kg in the skin layer and 0.14 W/kg in the fat layer. This is because skin has a lower depth of penetration than fat due to its greater conductivity value. At a frequency of 4.06 GHz, the skin conductivity is 2.38 S/m, and the fat conductivity is 0.512 S/m.

**Figure 7:**
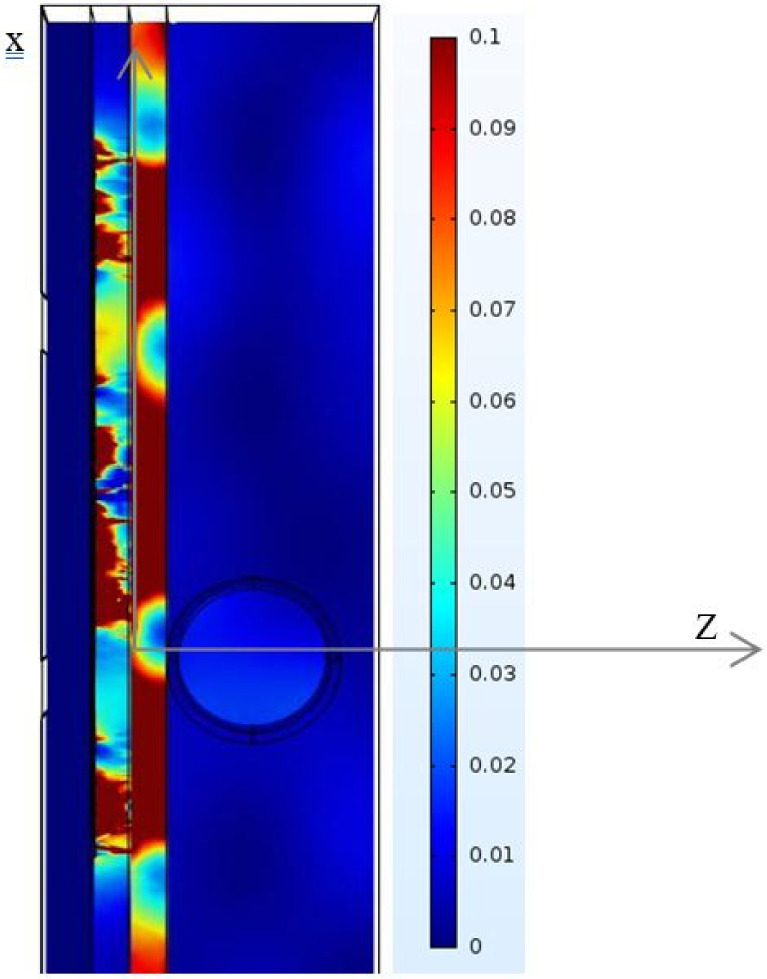
SAR distributions in the modeled phantom.

The other tissue constituents, such as blood, muscle, and blood vessel wall, present higher conductivities, which are 4.2, 3.07, and 2.66 S/m, respectively; it is very difficult for an EM field to pass through these constituents. Conductivity and permittivity are important parameters in mathematical models to predict the location and strength of an electrical generator in biological tissue [37,38]. Figure 7 shows a color map of the distribution of local SAR values, computed in a transversal section plan through the noninvasive sensor and correspondingly through the forearm tissue model. The map does not necessarily contain the highest SAR value inside the tissue. At a frequency of 4.06 GHz, the thickness values of skin and fat become significant in terms of wavelength, and reflection phenomena occur.

**Figure 8:**
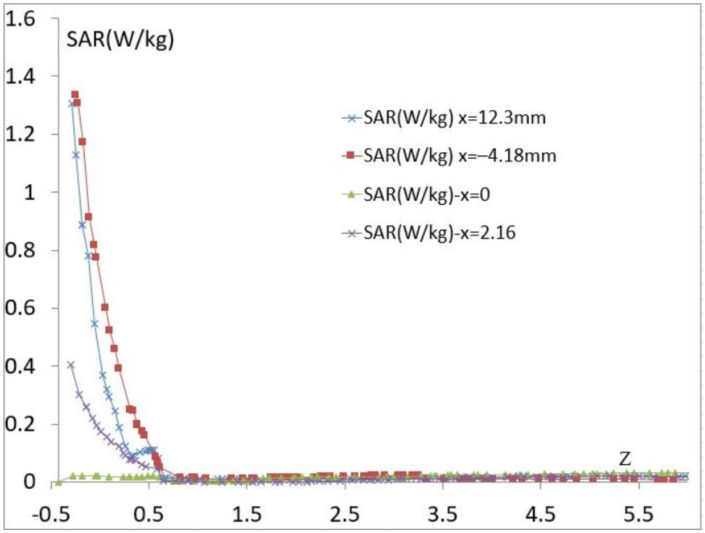
The calculated SAR vs. Z for the proposed model of a thin person.

The SAR can change from one person to another based on its tissue distribution and biological composition. The study of the local SAR on the tissue model of a thin person’s forearm is presented in Figure 8, and we notice that the local SAR in skin decreases in the same way as it does in the model with fat. This shows that skin behaves as a shield. Whatever the used tissue model is, the maximum of the SAR value is less than 1.4 W/kg, and the sensor meets the standard and it is comparable to some Industrial smartphones, indeed, some mobile phone brands exhibit an average SAR between 0.18 and 1.75 W/kg.

## 6. Conclusions

This paper presents a simulation study for the identification of the impact of a vein’s radius on the resonant microwave sensor. Furthermore, our sensor was SAR analyzed. Biological tissue models of an area from the forearm of a thin and overweight person were selected as a case study and were simulated. The simulation results show that the resonance frequency changes due to blood permittivity variation by a step of 0.03 are constant and equal to 0.01 GHz for three values of different radii of the vein. The maximum SAR value is equal to 1.23 W/kg, which is considered as the reference; the average SAR value is always lower than 1.23 W/kg. More studies should be conducted to calibrate the sensor according to each diabetic patient because of tissue heterogeneity.

## Data Availability

Not applicable.
